# Insights into Multifunctional Nanoparticle-Based Drug Delivery Systems for Glioblastoma Treatment

**DOI:** 10.3390/molecules26082262

**Published:** 2021-04-14

**Authors:** Mohd Khan, Subuhi Sherwani, Saif Khan, Sultan Alouffi, Mohammad Alam, Khalid Al-Motair, Shahper Khan

**Affiliations:** 1 Department of Chemistry, College of Sciences, University of Ha’il, Ha’il 2440, Saudi Arabia; 2 Molecular Diagnostic and Personalised Therapeutics Unit, University of Ha’il, Ha’il 2440, Saudi Arabia; s.alouffi@uoh.edu.sa (S.A.); k.almutier@uoh.edu.sa (K.A.-M.); 3 Department of Biology, College of Sciences, University of Ha’il, Ha’il 2440, Saudi Arabia; s.sherwani@uoh.edu.sa (S.S.); j.alam@uoh.edu.sa (M.A.); 4 Department of Basic Dental and Medical Sciences, College of Dentistry, University of Ha’il, Ha’il 2440, Saudi Arabia; sf.khan@uoh.edu.sa; 5 Department of Clinical Laboratory Sciences, College of Applied Medical Sciences, University of Ha’il, Ha’il 2440, Saudi Arabia; 6 Interdisciplinary Nanotechnology Centre, Aligarh Muslim University, Aligarh 202002, U.P., India; shahper01@gmail.com

**Keywords:** glioblastoma, polymeric nanoparticles, nanotherapeutic, blood–brain barrier, multifunctional, multicore

## Abstract

Glioblastoma (GB) is an aggressive cancer with high microvascular proliferation, resulting in accelerated invasion and diffused infiltration into the surrounding brain tissues with very low survival rates. Treatment options are often multimodal, such as surgical resection with concurrent radiotherapy and chemotherapy. The development of resistance of tumor cells to radiation in the areas of hypoxia decreases the efficiency of such treatments. Additionally, the difficulty of ensuring drugs effectively cross the natural blood–brain barrier (BBB) substantially reduces treatment efficiency. These conditions concomitantly limit the efficacy of standard chemotherapeutic agents available for GB. Indeed, there is an urgent need of a multifunctional drug vehicle system that has potential to transport anticancer drugs efficiently to the target and can successfully cross the BBB. In this review, we summarize some nanoparticle (NP)-based therapeutics attached to GB cells with antigens and membrane receptors for site-directed drug targeting. Such multicore drug delivery systems are potentially biodegradable, site-directed, nontoxic to normal cells and offer long-lasting therapeutic effects against brain cancer. These models could have better therapeutic potential for GB as well as efficient drug delivery reaching the tumor milieu. The goal of this article is to provide key considerations and a better understanding of the development of nanotherapeutics with good targetability and better tolerability in the fight against GB.

## 1. Introduction

Cancer is a pervasive and fast-growing disease, characterized by unchecked proliferation of cells. Various contributory factors in a modern lifestyle including risk factors, an improvement in general health rising from better health care facilities and an increasing life span have contributed to increasing incidence of cancers. Despite major developments in the fields of cancer research, including early detection and diagnosis and a multitude of treatment strategies, cancer is a leading cause of death globally.

Some cancers are biologically more aggressive in humans and characterized by fast development and poor patient prognosis. Glioma is a term used for glial cell brain tumors, i.e., of astrocytes, oligodendrocytes, microglia and ependymal cells. Glioblastoma multiforme (GBM), also known as glioblastoma (GB), is the commonest primary and fatal brain tumor [1]. In spite of medical advancements including aggressive surgical intervention, radiation and chemotherapies, over 15,000 new cases of GB are diagnosed each year in the United States of America [2] with a median survival rate of 14.6 months [2]. GBs are extremely aggressive vascularized tumors due to their invasive capacity, which in turn is associated with treatment resistance, recurrence and overall poor survival. Clinical symptoms are based on tumor size and location and may varyingly include an array of symptoms such as headache, dizziness, nausea, disorientation, seizures, difficulties with speech, change in personality and focal neurological deficits. The tumor is generally located in the cerebral hemispheres of the brain, although cases have also been reported to occur in the cerebellum, brainstem and spinal cord [3,4]. The complicated oncogenesis of gliomas presents various barriers which prevent drugs from reaching the site of the tumor. A major barrier for brain tumor therapy includes the blood–brain barrier (BBB), which protects the brain from exposure to dangerous substances in the blood and serves as an anatomic and metabolic barrier preventing the transport of molecules delivered into the brain.

In efforts to counter the disease, various new treatment modalities and therapeutics including novel anticancer compounds have been developed [5,6]. Nanoparticle (NP)-based treatment strategies for cancer which include drug delivery and targeted therapies can ensure precise targeting of cancer tissue with minimal side effects [7,8]. Importantly, due to their biological nature, these drug delivery systems can easily cross cell barriers and the BBB [9,10]. However, some issues associated with such anticancer drugs include solubility, kinetic stability and toxicity effects, as well as attainment of the desired concentration for optimum efficacy [11,12]. In light of this, several research studies were conducted to examine different NPs (polymers, liposomes, molecules, proteins and nucleic acids) which showed optimal outcomes with increasing efficacy and reduced toxicity [10,13]. Extensive research and clinical trials have been conducted for different nanomedicines to solve the impairments of drug delivery for GB.

Current research is focused on addressing challenges in new drug delivery approaches to improve treatment in clinical settings. We review some of the new nanotechnology-based approaches for drug delivery challenges to the brain, giving insight into those methods that are applicable to GB therapy.

## 2. Glioblastoma Treatment and Challenges

The GB microenvironment is characterized by tumor cells that produce factors that stimulate blood vessel formation to provide an uninterrupted supply of oxygen and nutrients to support growth and proliferation through continuous division. There is a substantial elevation of vascular endothelial growth factor activity, leading to high microvascular proliferation, resulting in invasion and diffused infiltration into the surrounding brain tissues. Due to the limitations of the available treatments, there is a need for better therapies for GB.

### 2.1. Treatment Strategies for Glioblastoma

#### 2.1.1. Initial Approach: Surgical Resection

The initial therapeutic approach for GB in adults is maximal safe surgical resection. This procedure also allows reduction in tumor size, histological diagnosis of tissue specimens and tissue genotyping. Studies indicate that surgical resection is linked with increased patient life expectancy. The standard procedure for glioblastoma includes adjuvant therapy, i.e., radiotherapy with chemotherapy with concomitant temozolomid (TMZ) [14]. TMZ, an oral alkylating agent, is a standard care option used to treat glioblastoma multiforme due to reported survival benefits in patients. However, the rising incidence of TMZ resistance due to persistence of cancer stem cell subpopulations, deregulation of signaling pathways, DNA repair and autophagy-related mechanisms have resulted in a rise in rates of treatment failure [15,16]. The development of resistance of tumor cells to radiation in areas with hypoxia decreases the efficiency of such treatments. Additionally, the difficulty of ascertaining that drugs successfully cross the natural BBB reduces treatment efficiency [17,18,19]. Advances in molecular genetics have led to the identification of the molecular basis of TMZ resistance in GBM. This has in turn spurred the development of new therapeutic strategies [20]. Inter- and intratumor heterogeneity and suppressed innate and adaptive immune mechanisms together with BBB-associated treatment resistance make GBM a lethal tumor type, which still remains a major challenge in GB-specific oncotherapeutics.

#### 2.1.2. Current Standard Treatment Options for Glioblastoma

Glioblastoma is a high-grade invasive brain tumor, characterized by peritumoral edema with inflammatory cells and matrix [21]. Chemotherapy and radiation together form the first line of treatment for this tumor. A lipophilic alkylating prodrug, TMZ, is administered orally in patients on a daily basis. However, patients still have poor overall prognosis and a high relapse rate [14,22]. Standard treatments remain ineffective for many reasons, such as the location of the tumor, which makes surgery difficult without damage to vital healthy brain cells. Hence, often surgery is rendered ineffective in removing all GB tumor cells, especially infiltrative cells. Other difficulties include the inability of chemotherapeutic drugs to cross the BBB and reach the tumor, as well as radio-resistant GB cells which are difficult to eliminate.

### 2.2. Challenges and Limitations in Drug Delivery across the BBB and BBTB

Challenges in the effective design and delivery of medicinal agents across the BBB is the major challenge to pharmaceutical companies for the treatment of central nervous system (CNS) diseases such as GBM [23,24]. An area of focus is the molecular and physical environment of the BBB, which presents the key challenge for drug transport, delivery and efficacy mechanisms across this barrier. The BBB is a selective semipermeable border with several components, which maintains brain homeostasis and guards neural tissue against exposure to foreign molecules. This dynamic system separates blood from neural tissue and is mainly composed of endothelial cells attached via tight junctions [25]. These gap junctions and proteins maintain a constant interstitial fluid environment by regulating the movement of molecules through the BBB [23,26]. The endothelial cells are partially covered with pericytes and basement membrane and further wrapped in astrocyte foot processes. These prevent the entry of all large and most small molecules. Entry of water, lipid-soluble molecules, amino acids and peptides to the interstitial fluid of the brain occurs through either simple or facilitated diffusion or via carrier transport [23,27]. Hence, the majority of small-molecule drugs and even larger-molecule drugs cannot cross the BBB [28,29].

Patients with high-grade brain tumors such as GB exhibit high permeability of the BBB due to disruptions resulting from leaky interendothelial tight junctions and the blood–brain tumor barrier (BBTB) [30,31,32]. One of the main reasons behind this is the downregulation of a protein, claudin-1, in high-grade GB, which is present in the tight junctions of endothelial cells. The disruption is not uniform, and some areas near the actively growing tumor edge with resident invasive tumor cells may still possess a relatively intact BBB [33]. An increased expression of proangiogenic factors such as vascular endothelial growth factor in brain tumors and existing hypoxic areas in high-grade GB causes cerebral microvascular perfusion and leakage [34,35]. An increased cerebral flow increases the pressure of interstitial fluid, which reduces movement of small-molecule drugs through the BBB to the tumor site [34]. Hence, BBB and BBTB pose major challenges in GB therapy by preventing the delivery of sufficient quantities of effective drugs to the tumor site [36,37], and alternative routes of delivery depending on the tumor type would greatly benefit research, with an overall improvement in the therapeutic efficacy of drugs. Selective targeting of tumors for drug delivery by overcoming the BBB may be an important therapeutic strategy for GB.

## 3. Nanotechnology-Based Treatment Strategies for Glioblastoma

Many delivery methods for therapeutic agents to the CNS have been developed, but most are considered invasive and do not reach the target specifically. Thus, current innovative approaches aim to improve targeted delivery as well as drug efficacy and safety. The biochemical disruption of the BBB membrane is one such strategy [38,39]. Nanoparticle-based therapies for GB help therapeutic drug delivery into the CNS by passing the BBB [10,40,41].

To date, many nanocarriers and micro-sized systems, such as poly-lactic-co-glycolic acid (PLGA), dendrimers, human serum albumin (HSA)-based nanoparticles (HSA-NPs), micelle polymers, carbon nanotubes, inorganic NPs, protein NPs, hybrid NPs, solid lipid NPs, niosomes, ROS-responsive glucose oxidase-loaded therapeutic nanoreactors, etc. have been utilized as biocompatible drug delivery systems. This has become possible through the adoption of a plethora of polymers, which are biomimetic, biodegradable, biocompatible and not capable of inducing an immunogenic response inside the host [42,43,44,45]. Currently, various targeting and delivery strategies are being explored in the design of different brain cancer-specific drugs, with efforts to enhance the therapeutic efficacy of the nanoparticles used. Some parameters for successful treatment through drug therapy require controlled release of the agent, in vivo stability and localized delivery of the drug. Nanotechnology products have revolutionized drug delivery systems in many cancers. Nanocomplexes consist of two main parts: the nanovehicle, i.e., the main carrier agent or vehicle; and the chemotherapeutic drug, which is located within a membrane or matrix and is either adsorbed, dissolved or dispersed into the tissue [38]. However, addressing issues such as toxicity, tissue specificity, concentration and side effects is still a work in progress. Nanotechnology provides a platform to meet and address these challenges.

Advanced research has been conducted in the last few years to develop a polyfunctional drug delivery system. In the last five years, important research has been conducted for NP-based drug delivery systems for GB which can cross the BBB (Table 1).

### 3.1. Stimuli-Responsive Nanocarriers for Glioblastoma

Lately, much development has been seen in the direction of stimuli-responsive nanoparticles, which could act as per the intrinsic physicochemical and pathological microenvironment of the brain cancer to ensure the specificity of drug delivery [63,64]. To date, a number of nanocarriers have been prepared with physicochemical changes in response to external stimuli, such as ultrasound, thermal, light and magnetic field, as well as internal stimuli, including pH, redox potential, hypoxia and enzymes [65,66,67,68]. The stimuli-responsive nanocarriers have been rationally designed and developed by considering different pathological profiles in normal tissues, intracellular compartments and the tumor microenvironment, to increase drug delivery specificity, efficacy and biological activities [69,70]. They could respond to stimuli in tumor microenvironments or inside cancer cells for site-specific drug delivery and accumulation, controlled drug release and the activation of bioactive molecules and targeting ligands, as well as size, charge and conformation conversion, leading to the execution of sensing and signaling, overcoming drug resistance and ensuring precision therapy [71].

Huang et al. [71] designed a novel hypoxia-responsive angiopep-2-lipid-poly(MIs)_n_ (ALP-(MIs)_n_) polyprodrug nanoparticle (NP) with hypoxic radiosensitization effects for targeted glioma therapy [72]. The drug was coloaded into ALP-(MIs)_n_ polyprodrug NPs to achieve chemotherapy and radiation synergistically. The obtained ALP-(MIs)_n_/drug complex was disintegrated to release the drug in the hypoxic conditions and showed significant inhibition of glioma tumor growth in combination with radiation therapy. Likewise, Yang et al. utilized a magnetic field for local area targeting which dramatically enhanced the drug accumulation in tumors and promoted the diagnosis (magnetically guided imaging) precision [73]. Hence, magnetically responsive systems provide precise prodrug delivery. Above all, the magnetic-guided targeting concept has shown great potential in glioma treatment.

Zhao et al. [74] exploited the acidic pH environment in gliomas by using a peptide, H7K(R2)2, as a targeting ligand. The H7K(R2)2-modified pH-sensitive liposomes containing doxorubicin (DOX-PSL-H7K(R2)2) were designed and tested for efficiency in glioma tumor cells and in mice bearing glioma tumors. The study reported a specific targeting effect triggered by an acidic pH in vitro experiment in C6 and U87-MG glioma cells. The antitumor activities of DOX-PSL-H7K(R2)2 were observed in C6 tumor-bearing mice and U87-MG orthotopic tumor-bearing nude mice in an in vivo study. The antiangiogenic activity of DOX-PSL-H7K(R2)2 has also been reported in mice bearing C6 tumor cells. The authors claimed that H7K(R2)2-modified pH-sensitive liposomes are a promising delivery tool for antitumor drug for gliomas.

Lee et al. [75] exploited the oxidative stimuli response to release camptothecin in gliomas. They prepared and characterized the nanoprodrug of camptothecin. The nanoprodrug was stimulated quickly by porcine liver esterase and, at a low rate, by hydrolytic degradation. Interestingly, the hydrolytic activation was insignificant prior to the oxidation, but showed remarkable increase after α-lipoic acid moiety oxidation, indicating an oxidative stimuli-responsive activation of the prodrug. The camptothecin nanoprodrug showed a remarkable inhibitory effect on the proliferation of U87-MG glioma cells.

Besides their therapeutic activation in response to stimuli, these nanocarriers have also attain superiority in terms of drug-loading capacity. These nanocarriers are often made of polymers/copolymers, which provide them with the common advantage of numerous functional groups on the polymer skeleton that can be chemically modified to form polymer–drug conjugates with higher drug-loading efficiency [76,77,78]. Recently, Arias et al. designed a novel theranostic nanomedicine with the ability to target the delivery of the gemcitabine under a magnetic field with a drug-loading efficiency of up to 93% [79]. In addition, considering the heterogeneity of brain tumors, the molecular imaging would be applied for screening the stimuli-responsive nanocarriers in patients, to predict and study the efficacy of the treatment [72]. Overall, the development of nanocarriers responding to external and internal stimuli in diseased regions would promote the advent of “magic bullets” for brain cancer precision diagnosis and therapy.

### 3.2. Transcytosable Nanomedicine

Transcytosis is a phenomenon of biomacromolecular transport across epithelial or endothelial barriers through vesicular translocation to preserve tissue metabolism and homeostasis [80]. Besides exploring paracellular transport, recent efforts have been dedicated to exploiting this active transcellular pathway of transcytosis. There are various types of transcytosis in which nanomedicine is relied upon for active luminal-to-abluminal transport in tumor and brain endothelium, which includes receptor-mediated transcytosis, absorptive-mediated transcytosis and bulk-phase or fluid-phase transcytosis.

Upon involving the endothelium, nanomaterials would in theory have the possibility to undergo transcytosis into the underlying tissue. Nevertheless, the crucial factors tuning nanomedicine properties could be multifaceted, including size, surface properties (e.g., charge, hydrophobicity, binding affinity and ligands/density/orientation), shape, physical nature, endosomal escape property and receptor turnover rate. Particularly, recent studies showed that nanomedicines without special features (e.g., charge and ligands) could enter into a tumor through transcytosis. As noted in several reports, transcytosis-capable nanomedicines hold emerging potential to facilitate deep penetration via consecutive transcytosis processes [81,82,83].

Thus, the manipulation of NPs for clinical applications depends on the extent of targeting of tumor cells via drug delivery. Some advantages of nanoparticulate drug delivery include the promotion of drug diffusion through the BBB, specific tumor-targeting mechanisms through enhanced permeability and retention effect, magnetic field gradient-induced diffusion of nanodrugs towards tumor and convection-enhanced delivery inducing the even distribution of GB drugs in the tumor stroma. The efficacy of various anti-GB drugs is also dependent on antitumor activities. Some of the mechanisms involved include the cellular internalization improving the efficacy of therapeutic drugs, increased effectiveness of radiotherapy due to a radio-sensitizing effect, activation of immune cells, destruction of angiogenic blood vessels and entry of antitumor drugs into GB by escaping the tumor surveillance system [10,84,85].

## 4. Novel Cancer Cell Membrane-Based Nanoparticles

Due to the emergence of a new generation of nanomaterials, and a greater understanding of nano–biological interactions, researchers are currently focusing on the development of next-generation nanoparticles with enhanced tissue, cellular and molecular functionalities [69]. Advances in molecular biology and nanotechnology have inspired scientists to use nanoparticles that mimic natural molecules. As is well documented, numerous cells are involved in the development and progression of cancer, e.g., red blood cells, leukocytes and platelets [86]; each playing a different role in the various stages. The properties of these cells, which are natural delivery vehicles, such as their structure, surface proteins and functionalities, have been included in the design and development of next-generation delivery platforms. The membrane specific functions of cells involved in cancer progression, such as rolling, extravasation, cell adhesion and chemotaxis, have inspired researchers to explore the cell membrane-based nanoparticles (CMBNPs) as carriers for tumor-targeted drug delivery [87,88]. Different types of cell membranes coating NPs may function differently, depending on the receptors and ligands attached. Next-generation NP design must draw on the realization and knowledge that natural components have purposely evolved for specific functions. Different cells exhibit unique properties dependent on the antigenic profile on their membranes. Thus, identification and understanding of individual membrane factors has enabled improvements in biomimetic features of synthetic platforms for advanced drug delivery specialized for specific cancers such as glioblastoma. Tumor antigens bound by membranes prime the immune system to identify and target cancers [89,90,91]. Hence, membrane-based NPs are a potential and versatile drug delivery system.

Glioblastoma is considered the most common and aggressive type of primary brain tumor, due to its unique position and environment in addition to its invasiveness, high proliferative index, immune evasion, genetic heterogeneity and genetic instability [14,92,93]. All these factors contribute to the limited efficacy of standard chemotherapeutic agents. The development of any new drug delivery systems must address issues of side effects and synthesis hurdles. Such drug delivery systems must be nontoxic, site-directed and exhibit long-lasting therapeutic effects in brain cancer models.

**Figure 1:**
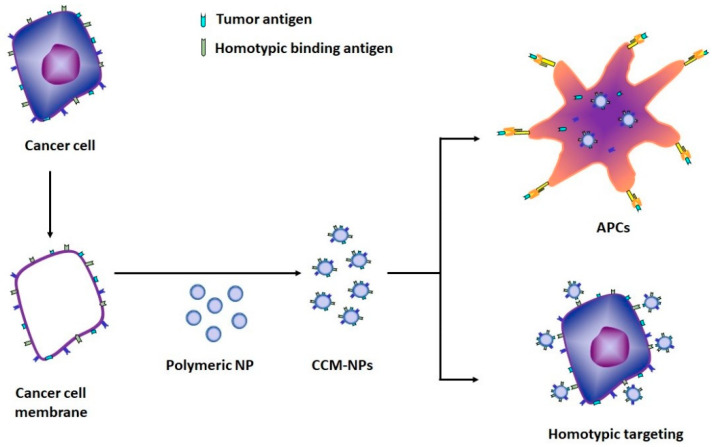
Schematic representation of the preparation of cancer cell membrane-coated nanoparticles (CCM-NPs) and two potential applications. APCs, antigen-presenting cells.

Among the many bioinspired strategies, this review also discusses the use of active cellular membrane materials for the fabrication of nanoparticles, representing the unique advantage of completely replicating cellular functions and surface antigenic diversity of cancer cells. This involves the biomimetic design of cellular membranes directly to nanoparticle form. The cancer cell membrane, with all associated antigens, is collected from source cancer cells and coated onto polymeric nanoparticle cores made of an organic polymer. The resultant cancer cell membrane-coated nanoparticles (CCM-NPs) have a high degree of self-recognition due to the transferred cell adhesion molecules and can homotypically target source cells or deliver tumor-associated antigens to antigen-presenting cells (APCs) (Figure 1).

Glioblastoma cells can be grown and harvested. By treating source cells with a hypotonic lysing buffer using an established procedure, purified cancer cell membrane can be collected [94]. CCM-NPs can be synthesized using a previously reported extrusion approach [95], and different core materials can be adapted and coated to achieve versatility. The rationale behind the CCM-NP carrier systems is that it presents an effective multiple membrane with antigens and specific targeting ligands. The platform facilitates the colocalization of multiple antigens with immunological adjuvants in a stabilized system, which can potentially lead to decreased off-target effects and immune activation. The membrane coating can be used to target source cells via homotypic binding in anticancer drug delivery systems.

Cancer cells are unconventional cells and have eccentric properties as compared to normal cells. Due to their proliferative ability, cancer cells can easily be derived through in vitro cell culture. Hence, it is not necessary to obtain cancer cells directly from the autologous plasma of patient or a donor. Homotypic metastatic cancer cells can reach distant tissues and establish secondary lesions due to membrane adhesion molecules [56,96]. Thus, cancer cell membranes can be used for surface functionalization and delivery of surface protein diversity via a complete membrane from the tumor cells onto the engineered NPs. Various cancer cell membrane-coated NPs are designed to mimic the inherent immune evasion strategy and adhesion properties of cancer cells. These properties can be manipulated for diagnosis, tumor targeting and therapy. Cancer cell membrane-cloaked upconversion nanoprobes have been developed to exhibit self-recognition, low immunogenicity and homologous targeting and binding effects [97].

Albumin is the most abundant plasma protein and is involved in the transportation of many in vivo molecules. It is also an important carrier of various drugs. Albumin NPs have garnered great attention as a chemotherapeutic delivery vehicle to tumors. Some benefits include biocompatibility, in vivo stability, nonimmunogenicity and targetability [98,99,100]. Furthermore, the ease of preparation of albumin NPs using conventional methods such as desolvation and emulsification add to the advantages. The presence of pre-existing functional groups including amines and carboxylates allow easy surface modification with many targeting ligands. An HSA-NP formulation with paclitaxel, known as Abraxane, was approved by the United States Food and Drug Administration for treating metastatic breast cancers [101,102].

We have established a pharmacokinetic model for anticancer drug delivery by investigating binding kinetics with serum albumin [103,104]. Binding significantly affects the apparent distribution of drug volume and the elimination rate and therapeutic effectivity of drugs. Recently, working in the direction of biocompatible nanoparticle-based anticancer therapeutics for treatment of drug-resistant cancers, a study using plasma concentration of human serum albumin found the fraction of free drug to be 18% greater for the B isomer than the N isomer (two conformers of HSA) [103,104,105]. Hence, the potential of HSA as a biomolecule which can be considered in membrane-based nanoparticle targeted drug delivery should be investigated further.

## 5. Fate of NPs in In Vivo Systems

The nano–bio microenvironment interface is the boundary between engineered nanomaterials and a biological system and represents a dynamic environment where the NP surface interacts with biological entities of the surroundings. The in vivo fate of NPs is determined by the interactions that occur at this interface. The physicochemical composition of NPs, including size, shape, surface area, charge, porosity and functionalization, actively contribute to the stability as well as interactions with biological components once NPs enter into the bloodstream [106]. These interactions also depend upon not only the characteristics of the nanoparticle but also the biological environment and any biomolecules in it. Studies indicate that NPs approximately 100 nm in size demonstrate longer half-lives in blood. Also, discoid particle shapes enhance the margination of blood vessel walls [107,108]. Such NP features improve NP stability and survival by avoiding clearance and interacting with the endothelium and increase the probability of NPs reaching the target tissue. Other studies found that the surface adsorption of serum proteins (e.g., albumin, opsonins, etc.) was reduced by neutral or negatively charged NPs [109,110]. Immune system and nano–bio interface interactions also occur during systemic circulation as well as in the target tissue.

NPs with an exterior coated with opsonin proteins undergo significant surface composition changes which mediate their interactions with other cells [111]. NPs coated with opsonin protein communicate the presence of a foreign entity for immediate clearance to the circulating macrophages. Alternatively, a NP with a negative surface charge or polymer coating minimizes opsonin protein binding, reducing chances of clearance by circulating cells and hence increasing chances of reaching the target site. Such strategies reduce interactions between cells and particles in the blood. Some common chemical surface functionalization methods include surface coating with chitons, polyethylene glycol and dextrans [112,113,114]. Similarly, adding proteins, such as CD45 and CD47, can help NPs to evade clearance [115].

Another class of molecules which mediate nano–bio interface interactions in vivo and can be exploited for nanotherapeutics are scavenger receptors, such as SR-A, SR-B1, CD36, and MARCO, found on many cell types such as macrophages and endothelial cells. These are known to facilitate the uptake of a variety of foreign and endogenous ligands. Cells with these ligands in the blood or tissue interact with NPs [116]. Some NPs bind to these receptors to mediate cellular uptake. An example is the improved targeting of ovarian and colorectal cancer cells via high levels of SR-B1 [117]. Similarly, uptake of silver NPs by macrophages is also mediated by SR-B1, while simultaneously inducing proinflammatory cytokine overexpression [118]. Ligand expression on NP surface for scavenger receptors may have beneficial effects such as accumulation in target sites. These receptors may also lead to some unfavorable consequences. Circulation time of NPs can be reduced by macrophage uptake. Also, NPs may affect scavenger receptor density on cell surfaces, leading to altered responses to endogenous ligands and accelerating disease progression. Scavenger receptors help to transform and differentiate macrophages into foam cells, which results in the acceleration of atherosclerosis [119]. Thus, nano–bio interactions may have both positive and negative outcomes for NPs. The fine-tuning of these interactions is crucial for the successful application of nanotherapeutics.

Another important function of NPs used in nanotherapeutics is the communication and stimulation of responses through immune cell interactions found in the disease state. The surface features of NPs provide the main features for this communication, as most immune cells are activated through binding with antigens present either on the surface of other cells or molecules [120]. Thus, NPs also serve as artificial APCs and present cell surface features that activate, stimulate or control immune cells or genes [121]. The biomimetic ability of NPs to mimic native APCs has enabled engagement with immune cells. For example, the dendritic cell-like characteristics mimicked by some NPs using nanotube morphology have shown stronger interactions with target immune cells [122]. An increase in the contact surface area of such particles improves visibility by other immune cells such as by T cells and even mediate important receptor–ligand interactions [123].

Thus, the addition of stimulatory and regulatory molecules on the surface of NPs facilitates communication with immune cells. NP moieties such as MHC peptides, CD80 and CD86 can bind to and stimulate T cells, leading to an expansion of cytotoxic T cells that infiltrate the tumor. These go on to increase regulatory T cells, which in turn downregulates an overactive immune response, as discussed earlier [124,125]. Therefore, specially designed NPs have the capacity to communicate with immune cells through the expression of molecules already existent on native immune cells. Such immunomodulation interactions can further trigger the priming of favorable immune responses for the disease.

## 6. Conclusions

It is a well-known fact that GB has high invasiveness, is immunologically evasive, has a high proliferative index and exhibits genetic heterogeneity and instability as well as occupying a unique intracranial environment with physio-anatomic barriers between the neural tissue and the tumor. These conditions result in the limited efficacy of current standard chemotherapeutic agents. Hence, there is a need for the development of multifunctional drug vehicles which can transport drugs efficiently to the target site, crossing the BBB. HSA-based polymeric NPs coated with cancer membrane proteins for specific targeting are potentially nontoxic to normal cells and exhibit site-directed delivery with long-lasting therapeutic effects against brain tumors. These multicore drug delivery models present efficient targeted in vivo drug delivery systems with minimal systemic toxicity. However, in order to translate the experimental studies to clinical trials, further investigations are necessary, particularly to optimize the drug concentrations that reach the targeted area for the best clinical outcome.

## Figures and Tables

**Table 1:** A summary of various NP-based drug delivery systems which can cross the blood–brain barrier for the potential treatment of glioblastoma.

Composition of NPs	Coating	Cell Lines	In Vitro Effects on Cell Lines	In Vivo Effects	Outcome	Ref.
PLGA	PTX/SPIO	U87MG	Cytotoxic	GB tumor progression substantially decreased in mice	Increased accumulation in tumor tissue	[46]
Mesoporous silica	DOX-PDA-NGR	C6	Cytotoxic	Increased survival in orthotopic glioma nude mice	Higher accumulation in intracranial tumorous tissue	[47]
Magnetite	Polyplex + BCNU	HGB	Cytotoxic	-	Effective uptake and internalization of BCNU-loaded Nano-co-plex in HGB cells	[48]
Cisplatin-Fe_3_O_4_ /Gd_2_O_3_	LF + RGD dimer	U87-MG	Cytotoxic	Survival rate of U87-Luc-bearing mice increased	Uptake by cancer cells and release of Fe^2+^, Fe^3+^	[49]
Au NP AuNRs@SiO_2_	RVG29; PEG	N2a	Cytotoxic, increased cellular uptake into neuronal cells	Efficient internalization into N2a cells and delay in tumor growth	Photothermal therapy crosses BBB via interaction between RVG29 and AchR	[50]
Iron oxide	NIR-fluorescent silica	U87-MG	Uptake by NP	Delineation of GB	Providing accurate delineation of tumor margins through tumor-associated macrophages	[51]
Liposome (thermoresponsive)	PTX	U-87 MG	Higher cytotoxicity against U-87 MG cells at 39 °C compared to 37 °C	-	Drug release rate was faster at hyperthermic conditions	[52]
Liposome	Cyclic peptide iRGD + siRNA	U87, GL-261	Cytotoxic for both cell lines and downregulation of PD-L1 and EGFR	Slower tumor growth and increased mouse survival	Radiation therapy primed GB for f(SLN)-iRGD:siRNA targeted EGFR and anti-PD-L therapy and led to slower tumor growth and enhanced mouse survival	[53]
Liposome + magnetic nanovectors	Temozolomide	U87- MG	Cytotoxic	-	TMZ-LMNVs + AMF group showed apoptotic and antiproliferative effects	[54]
Liposome	Ursolic acid + EGCG + MAN	C6, C6-GSCs	Anti-proliferative effect	Killing of C6 and C6-GSCs and the survival time of mice increased	Ursolic acids arrested G2 and EGCG could arrest G0/G1 phases of the cell cycle, caused stronger antiproliferative effects	[55]
Liposome	ICG+ GB membrane proteins	C6	Cytotoxic	Superior homotypic targeting ability of BLIPO-ICG to glioma caused cell apoptosis	High accumulation in the brain tumor	[56]
Dendrimer	RGDyC-mPEG-PAMAM-arsenic trioxide (ATO)	C6	Cytotoxic	Cell apoptosis in tumor tissue	RGDyC-mPEG-PAMAM/ATO arrested the cell cycle in G2–M	[57]
Albumin	Paclitaxel and fenretinide	U87	Cytotoxic	Decrease in tumor growth delay with increased survival rate	Albumin-based drug delivery had enhanced tumor accumulation and intratumoral infiltration	[58]
Methylene blue oleate salt-loaded polymeric NP	Methylene blue	U87, T98G	U87 and T98G cell inhibition	Effective BBB crossing of NPs	Release of drug into GB tumor	[59]
Micelle (PEtOz-SS-PCL)	DOX	C6	Cytotoxic	Prolonged survival times in glioma bearing mice	Therapeutic efficacy for glioma, due to the smallest nanosize that overcame the BBB	[60]
Micelle	BCNU + T7 peptide	U87	Cytotoxic	Apoptosis was observed inside the tumor site	T7–PEG–PLGA/Cou6 NPs observed in tumor and increased drug efficacy	[61]
Fa-PEG-PCL	Luteolin	GL261	Cytotoxic	Significant antitumor effect and increased survival of mice with GL261 tumor	Luteolin/FaPEG-PCL NPs inhibited the neovascularization of GL261 glioma that may inhibit tumor growth	[62]

PLGA, poly(lactic-co-glycolic acid); PTX, paclitaxel; SPIO, superparamagnetic iron oxide nanoparticles; U87/U87-MG/C6, glioblastoma cancer cell line; GB, glioblastoma; DOX, doxorubicin; PDA, polydopamine; NGR, Asn-Gly-Arg; BCNU, carmustine; HGB, human glioblastoma; Fe_3_O_4_, iron (II, III) oxide, Gd_2_O_3_, gadolinium oxide; LF, lactoferrin; RGD2, RGD dimers; RVG-PEG-AuNRs@SiO_2_ (rabies virus-mimetic silica-coated gold nanorods); AchR (nicotinic acetylcholine receptor); C6-GSCs, glioblastoma stem cells; GBM, glioblastoma multiforme; RVG, rabies virus glycoprotein; RVG-29, 29-residue peptide derived from RVG; PEG, polyethylene glycol; N2 cells, neuroblastoma cell line; BBB, blood–brain barrier; PEtOz-SS-PCL, copolymer poly (2-ethyl-2-oxazoline)-b-poly (ε-caprolactone); C6-Luci cells, modified C6 cells which can express luciferase; Fa-PEG-PCL, folic acid-modified poly(ethylene glycol)-poly(e-caprolactone); GL261, glioma cell line; cyclic peptide iRGD, 9-amino acid (sequence: CRGDKGPDC) cyclic peptide; siRNA, small interfering RNA.
